# Crop to wild introgression in lettuce: following the fate of crop genome segments in backcross populations

**DOI:** 10.1186/1471-2229-12-43

**Published:** 2012-03-26

**Authors:** Brigitte Uwimana, Marinus JM Smulders, Danny AP Hooftman, Yorike Hartman, Peter H van Tienderen, Johannes Jansen, Leah K McHale, Richard W Michelmore, Richard GF Visser, Clemens CM van de Wiel

**Affiliations:** 1 Wageningen UR Plant Breeding, Postbus 386, 6700AJ Wageningen, the Netherlands; 2 Wageningen UR Plant Breeding, Postbus 16, 6700AA Wageningen, the Netherlands; 3 Centre for Ecology & Hydrology, Maclean Building, Benson Lane, Crowmarsh Gifford, Wallingford, Oxfordshire, OX10 8BB, UK; 4 Institute for Biodiversity and Ecosystem Dynamics, University of Amsterdam, Postbus 94248, 1090 GE Amsterdam, the Netherlands; 5 Wageningen UR Plant Biometris, Postbus 100, 6700AC Wageningen, the Netherlands; 6 Department of Horticulture and Crop Science, The Ohio State University, Columbus, OH 43210, USA; 7 Genome Center and Department of Plant Sciences, University of California Davis, Davis, CA 95616-8816, USA

## Abstract

**Background:**

After crop-wild hybridization, some of the crop genomic segments may become established in wild populations through selfing of the hybrids or through backcrosses to the wild parent. This constitutes a possible route through which crop (trans)genes could become established in natural populations. The likelihood of introgression of transgenes will not only be determined by fitness effects from the transgene itself but also by the crop genes linked to it. Although lettuce is generally regarded as self-pollinating, outbreeding does occur at a low frequency. Backcrossing to wild lettuce is a likely pathway to introgression along with selfing, due to the high frequency of wild individuals relative to the rarely occurring crop-wild hybrids. To test the effect of backcrossing on the vigour of inter-specific hybrids, *Lactuca serriola*, the closest wild relative of cultivated lettuce, was crossed with *L. sativa *and the F_1 _hybrid was backcrossed to *L. serriola *to generate BC_1 _and BC_2 _populations. Experiments were conducted on progeny from selfed plants of the backcrossing families (BC_1_S_1 _and BC_2_S_1_). Plant vigour of these two backcrossing populations was determined in the greenhouse under non-stress and abiotic stress conditions (salinity, drought, and nutrient deficiency).

**Results:**

Despite the decreasing contribution of crop genomic blocks in the backcross populations, the BC_1_S_1 _and BC_2_S_1 _hybrids were characterized by a substantial genetic variation under both non-stress and stress conditions. Hybrids were identified that performed equally or better than the wild genotypes, indicating that two backcrossing events did not eliminate the effect of the crop genomic segments that contributed to the vigour of the BC_1 _and BC_2 _hybrids. QTLs for plant vigour under non-stress and the various stress conditions were detected in the two populations with positive as well as negative effects from the crop.

**Conclusion:**

As it was shown that the crop contributed QTLs with either a positive or a negative effect on plant vigour, we hypothesize that genomic regions exist where transgenes could preferentially be located in order to mitigate their persistence in natural populations through genetic hitchhiking.

## Background

One of the debated ecological risks associated with the commercial cultivation of genetically modified crop varieties is the possibility of introgression of transgenes from crops to their wild relatives through hybridization. Possible adverse consequences of introgression would be an increase in the weediness of the wild relatives in agricultural areas, genetic erosion in wild relatives, or the invasion of new habitats by crop-wild transgenic lineages [1-4]. Where crops and their compatible wild relatives coexist, hybridization between the two is likely [5,6]. Therefore, the outcome of hybridization between crops and their wild relatives has been the subject of several research studies, using either transgenic or conventional crop varieties [7-10].

The net effect of crop-wild hybridization in terms of fitness may be negative, for instance if crop genes reduce the competitive ability under natural conditions, or positive, if hybrids inherit combinations of additive positive traits from the crop and the wild parents [11]. If hybrids are viable and fertile, hybridization can result in a swarm of hybrids in which crop and wild genomes interactively define the hybrid phenotypes. From the F_1 _progeny onwards, crop alleles can be fixed through selfing or through backcrossing to the wild parent followed by selfing, or through combinations of these, depending on the breeding system of the species. Natural selection will purge maladapted genotypes, leaving those genotypes with similar or higher net fitness as the wild parent in the natural habitat of the wild taxon, or with broadened adaptation as a result of transgressive segregation [12,13].

Initially, any crop gene in a hybrid plant will be in a chromosome segment comprising the gene itself and other crop genes linked with it, and the fitness effect will depend on the overall effect of the whole chromosome segment [14]. In subsequent generations, these haplotypes will gradually be broken up through recombination, but loci at short genetic distances from each other may remain linked for many generations [15]. In the course of crop allele fixation, a gene that confers a selective advantage may be introgressed, but it will do so along with other loci tightly linked to it, which may also have an effect on fitness. A gene may also be selected against, if it is linked to a deleterious gene [16-18]. It is within such a context that the dynamics of the process of introgression from crops to wild relatives constitute a baseline for understanding the effects of transgene escape and fixation into wild taxa [7,19].

We have initiated a study in which we follow the genetic process of introgression from cultivated lettuce (*Lactuca sativa *L.) to its wild relative prickly lettuce (*Lactuca serriola *L.). The two species readily hybridize, resulting in viable and fertile hybrids [20], hence representing a typical crop-weed complex. Despite the limited outcrossing rate in the two species [21,22], through population-genetic means we have identified crop-wild hybrid plants among natural populations of *L. serriola *which are expected to be a result of spontaneous gene flow between the two species [23].

In a previous study we have explored the genetic basis of hybrid vigour in an F_2 _population resulting from a synthetic cross between cultivated *L. serriola *and *L. sativa *[Uwimana *et al. *submitted]. We mapped QTLs for plant vigour, which co-localized in a small number of chromosome regions, with genetic variation explained by both additive main effect and epistatic QTL effect. After hybridization, the crop genomic segments will be established in the wild background or eliminated by selection either through selfing of the hybrids or through backcrossing to the predominant wild plants, or a combination of the two processes. Selfing generations after a single hybridization event between the crop and the wild parents are characterized by crop genomic segments constituting an average of 50% of the hybrid genome. In contrast, every backcross to the wild parent decreases the crop genome content by half, while the crop genome segments become smaller through recombination (Additional file 1: Figure S1). In this way, crop segments that contribute to the vigour and fitness of the hybrids get introgressed with a decreasing number of hitchhiking loci with each backcross generation. Therefore, the fitness effects of a transgene in the context of its genomic location will differ in the selfing and backcrossing pathways.

Studies on crop-wild hybrids are usually conducted on selfing generations of the hybrids [7,8,24,25] and rarely on backcross populations [26], hence overlooking a significant pathway in the crop-to-wild introgression process. In this study we follow up the crop-weed complex of *L. sativa *and *L. serriola *in a marker-assisted introgression study, and we focus on BC_1 _and BC_2 _generations in which *L. serriola *was the recurrent parent, hence mimicking the introgression process from crops to wild relatives through repeated backcrosses with wild populations. Abiotic stresses constitute major selection factors that impact the frequency of specific crop segments in subsequent generations [27-29]. Moreover, considerable effort is presently put into developing transgenic varieties capable of withstanding abiotic stress factors [30,31]. Therefore, the two hybrid populations were tested under three abiotic stress conditions, namely drought, salinity and nutrient deficiency. We aimed at obtaining answers to the following questions: (1) Do the backcross generations exhibit transgressive segregation for vigour? (2) Are the vigour QTL regions that were identified in the selfing pathway (F_2 _population) also detected in the backcross populations? (3) How does the contribution of crop alleles to the vigour of the hybrids change with the increasing proportion of wild genetic background?

## Results

### Phenotypic variance among the hybrid families

Backcrossing made the hybrid plants morphologically very similar to their wild parent, *L. serriola*. The BC_1_S_1 _and BC_2_S_1 _families showed a wide range of means for the vigour traits under stress and non-stress conditions (Table 1). Vigour depended on the backcross families and varied between the treatments in the two hybrid populations as revealed by the significance of GxE (*P*_genotype × treatment _< 0.001 for all traits). Some trait-treatment combinations, such as plant height under all the treatments and dry weight under control and drought conditions, showed transgressive segregation over the two parents (Table 1). For all traits and in both backcross generations the mean of the wild parent *L. serriola *was lower than the maximum mean of the hybrid families. In spite of a second generation of backcrossing from BC_1 _to BC_2_, for each trait-treatment combination individual BC_1_S_1 _and BC_2_S_1 _plants and families stood out that performed better than the two wild genotypes (*L. serriola*/Eys and *L. serriola *acc. UC96US23, Table 1 and Additional file 1: Figures S3 and Figure S4), indicating that the BC_2 _plants still contained crop genomic segments which contributed positively to their vigour.

**Table 1:** Parental means and mean, minimum and maximum values and heritability of the BC_1_S_1 _and BC_2_S_1 _families for vigour traits under non-stress, drought, salinity and nutrient deficiency conditions

		*L. serriola*	*L. sativa*	Hybrid families			
			
Trait	Treatment			Mean	Min	Max	**H**^**2**^
				**BC_1_S_1 _families**			

Plant height (cm)	Control-D^1^	31.42	23.52	30.87	27.43	36.19	0.86

	Drought	16.58	13.19	16.05	13.53	18.53	0.74

	Control- SN	33.36	17.95	28.75	22.39	42.64	0.95

	Salt	16.72	14.70	17.18	13.40	24.93	0.95

	Nutrient deficiency	10.57	8.75	10.07	7.88	13.35	0.86

Fresh weight (g)	Control-D	39.20	68.11	46.56	25.25	62.26	0.87

	Drought	6.48	8.01	6.46	5.40	8.14	0.48

	Control- SN	25.52	39.15	27.24	21.76	32.27	0.79

	Salt	8.40	20.13	10.85	7.90	14.35	0.69

	Nutrient deficiency	2.60	4.82	3.16	2.46	3.92	0.44

Dry weight (g)	Control-D	2.42	3.14	2.87	1.51	4.09	0.90

	Drought	1.15	1.38	1.16	0.93	1.41	0.80

	Control-NS	2.01	2.56	2.13	1.61	2.75	0.75

	Salt	0.84	1.79	1.07	0.74	1.43	0.59

	Nutrient deficiency	0.50	0.90	0.61	0.42	0.86	0.62

Relative moisture	Control-D	93.80	95.41	93.89	93.07	94.83	0.75

content (%)	Drought	81.97	82.56	81.77	79.26	84.74	0.69

	Control-SN	92.11	93.50	92.24	91.25	93.21	0.64

	Salt	90.00	91.18	90.18	89.23	91.24	0.67

	Nutrient deficiency	81.03^ns^	81.85	80.77	77.84	85.09	0.90

				BC_2_S_1 _families			

Plant height (cm)	Control-D^1^	27.93	23.07	29.17	24.09	37.01	0.85

	Drought	14.02	12.32	14.32	12.39	17.75	0.77

	Control- SN	21.02	16.63	21.51	17.51	28.04	0.80

	Salt	16.54	13.62	16.54	13.01	22.2	0.84

	Nutrient deficiency	11.62	10.07	11.33	9.69	14.05	0.43

Fresh weight (g)	Control-D	27.21	67.25	38.59	23.38	54.89	0.73

	Drought	5.24^ns^	5.46	4.55	3.31	6.32	0.37

	Control- SN	13.70	31.32	17.64	13.19	26.39	0.72

	Salt	9.31	17.87	10.35	7.27	13.36	0.63

	Nutrient deficiency	4.87	7.14	5.34	4.26	7.65	0.59

Dry weight (g)	Control-D	2.08	3.34	2.76	1.81	3.95	0.80

	Drought	1.12^ns^	1.22	1.06	0.88	1.27	0.50

	Control-NS	1.03	1.88	1.31	0.96	1.94	0.71

	Salt	0.84	1.29	0.92	0.65	1.21	0.61

	Nutrient deficiency	0.68	0.83	0.71	0.50	1.16	0.76

Relative moisture	Control-D	92.28	95.06	92.86	92.06	94.03	0.78

content (%)	Drought	77.94^ns^	77.53	76.12	71.72	80.39	0.77

	Control-SN	92.52	94.06	92.61	91.79	93.79	0.73

	Salt	90.95	92.85	91.24	89.92	92.36	0.76

	Nutrient deficiency	85.88	88.26	86.70	84.49	89.48	0.79

1 Control-D: the control treatment of the drought experiment, Control-SN: the control treatment of the salt-nutrient experiment

Genetic variation as measured through the broad sense heritability of family means of the traits ranged from 0.44 to 0.95 in the BC_1 _experiments, showing that a substantial part of the phenotypic variation was due to genetic factors (Table 1). In the drought experiment, heritability was lower in the drought treatment than in the control for all traits. In the salt-nutrient experiment, the heritability was lower in the salt and nutrient deficiency treatment than in the control for plant height, fresh weight and dry weight, but the heritability was higher for relative moisture content, with a greater difference in the nutrient deficiency treatment (0.90 compared to 0.64 in the control).

In the BC_2 _population, heritability of the traits among BC_2_S_1 _families ranged from 0.43 to 0.85, which is comparable to the range found in the BC_1 _population (Table 1). Also in line with the BC_1 _population, the heritability was lower in the drought treatment than in the control for all the traits in the BC_2 _population. In the salt-nutrient experiment, heritability was lower in the salt treatment than in the control for fresh weight and dry weight, while it was slightly higher than the control for plant height and relative moisture content. In the same experiment, heritability was considerably lower under nutrient deficiency conditions as compared to the control for plant height, with 0.85 under control and 0.43 under nutrient deficiency conditions.

### Allelic composition of the hybrids and linkage maps

BC_1 _individuals contained on average 26% of the crop genome with individual plants ranging from 11% to 39%. The population was characterized by long crop genomic segments in a heterozygous state which sometimes spanned all the markers of a whole linkage group (Additional file 1: Figure S2A). One additional backcross to the wild parent resulted in a reduction of the crop genome content to 14%, varying among BC_2 _individuals both in segment size and proportion, ranging from 3% to 29% (Additional file 1: Figure S2B).

**Figure 1:**
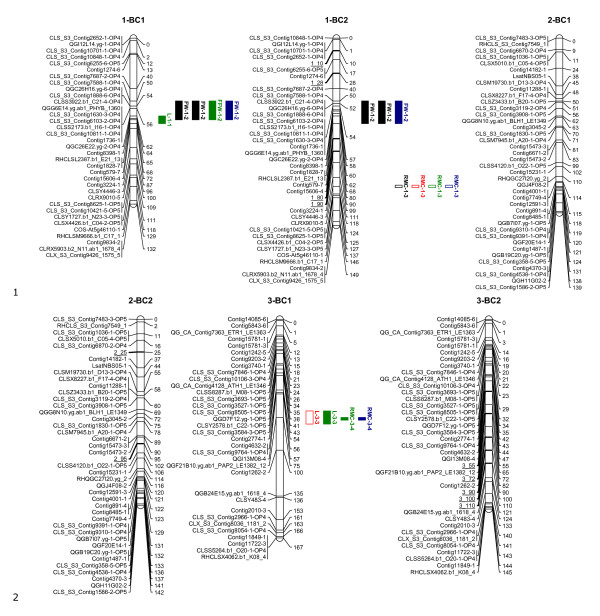
**Linkage maps of the BC_1 _and BC_2 _populations based on 100 and 458 individuals respectively: Linkage groups 1-3**. Markers are shown on the left of the bar and their positions on the right in cM. The added virtual markers on the BC_2 _map with missing scores are underlined. Vigour QTLs as mapped in BC_1_S_1 _and BC_2_S_1 _families under non-stress (black), drought (red), salt (blue) and nutrient deficiency (green) conditions are shown next to the marker positions. Open QTL block indicates a positive additive effect for the wild allele, and closed QTL block indicates a positive additive effect for the crop allele. Trait abbreviations: L: plant length, FW: fresh weight, DW: dry weight, RMC: relative moisture content.

**Figure 2:**
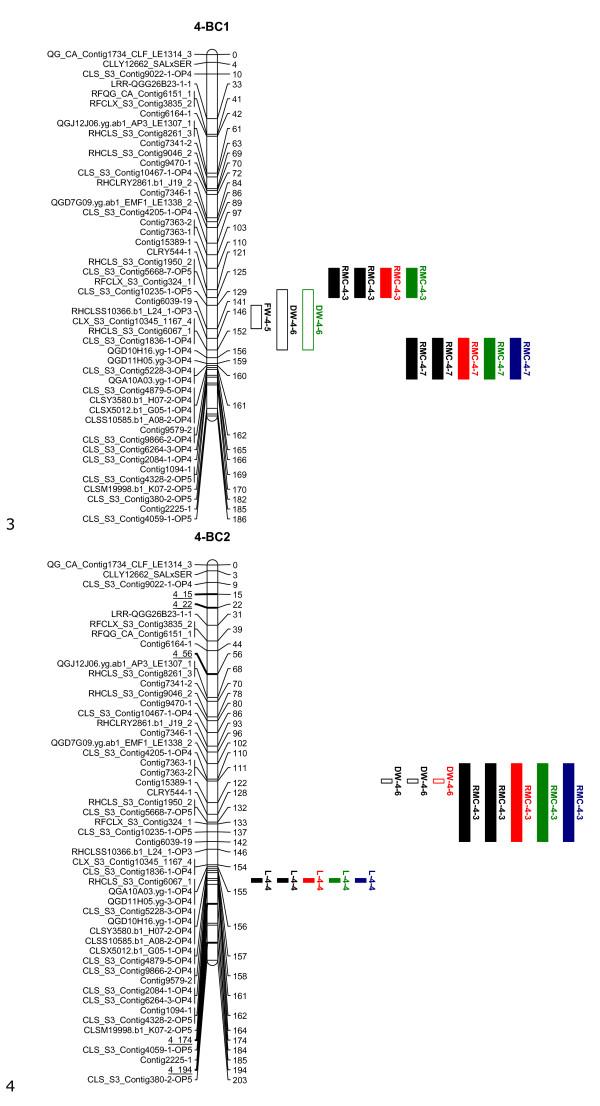
**Linkage maps of the BC_1_ and BC_2_ populations based on 100 and 458 individuals, respectively: Linkage group 4**. Markers are shown on the left of the bar and their positions on the right in cM. The added virtual markers on the BC2 map with missing scores are underlined. Vigour QTLs as mapped in BC_1_S_1_ and BC_2_S_1_ families under non-stress (black), drought (red), salt (blue) and nutrient deficiency (green) conditions are shown next to the marker positions. Open QTL block indicates a positive additive effect for the wild allele, and closed QTL block indicates a positive additive effect for the crop allele. Trait abbreviations: L: plant length, FW: fresh weight, DW: dry weight, RMC: relative moisture content.

**Figure 3:**
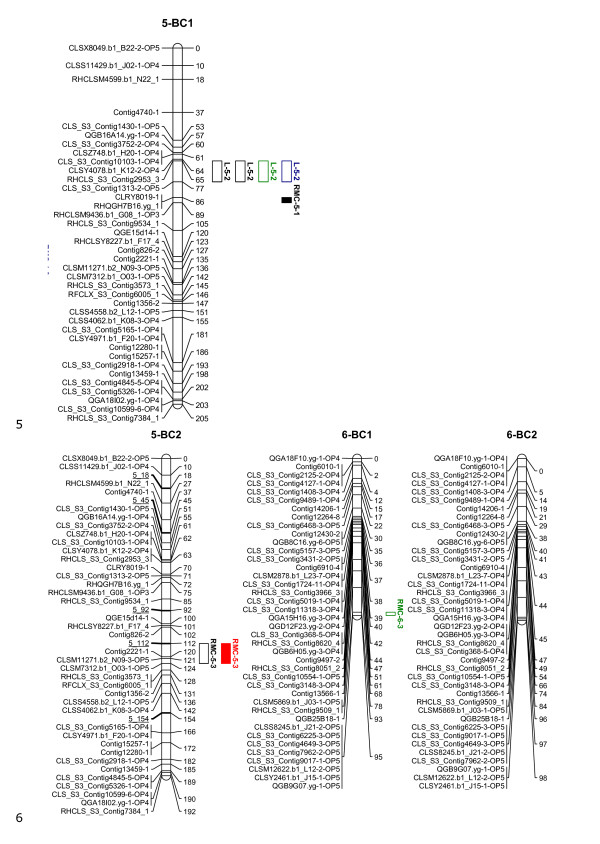
**Linkage maps of the BC_1_ and BC_2_ populations based on 100 and 458 individuals, respectively: Linkage groups 5-6**. Markers are shown on the left of the bar and their positions on the right in cM. The added virtual markers on the BC_2_ map with missing scores are underlined. Vigour QTLs as mapped in BC_1_S_1_ and BC_2_S_1_ families under non-stress (black), drought (red), salt (blue) and nutrient deficiency (green) conditions are shown next to the marker positions. Open QTL block indicates a positive additive effect for the wild allele, and closed QTL block indicates a positive additive effect for the crop allele. Trait abbreviations: L: plant length, FW: fresh weight, DW: dry weight, RMC: relative moisture content.

**Figure 4:**
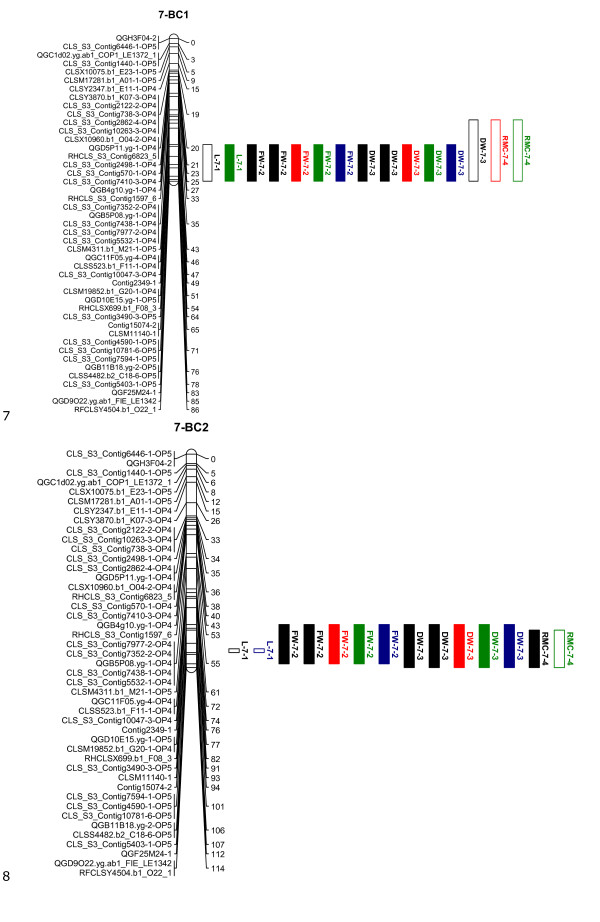
**Linkage maps of the BC_1_ and BC_2_ populations based on 100 and 458 individuals, respectively: Linkage group 7**. Markers are shown on the left of the bar and their positions on the right in cM. The added virtual markers on the BC_2_ map with missing scores are underlined. Vigour QTLs as mapped in BC_1_S_1_ and BC2S1 families under non-stress (black), drought (red), salt (blue) and nutrient deficiency (green) conditions are shown next to the marker positions. Open QTL block indicates a positive additive effect for the wild allele, and closed QTL block indicates a positive additive effect for the crop allele. Trait abbreviations: L: plant length, FW: fresh weight, DW: dry weight, RMC: relative moisture content.

**Figure 5:**
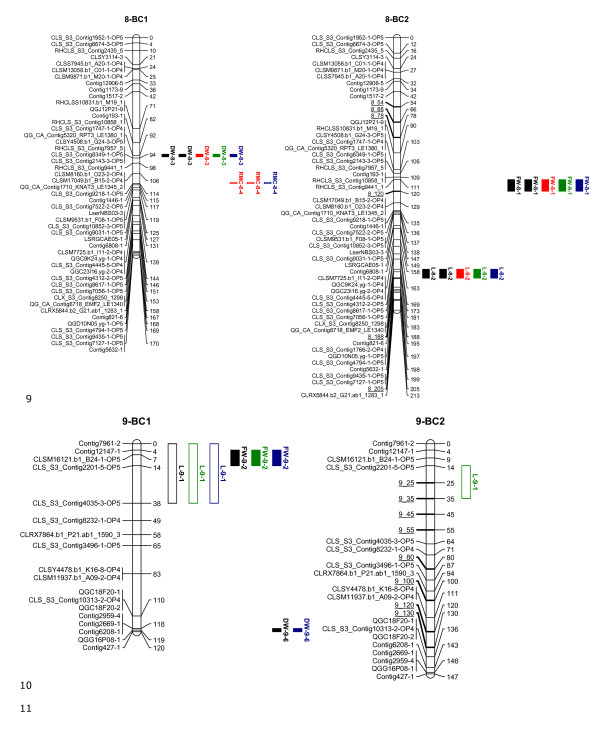
**Linkage maps of the BC_1_ and BC_2_ populations based on 100 and 458 individuals, respectively: Linkage groups 8-9**. Markers are shown on the left of the bar and their positions on the right in cM. The added virtual markers on the BC_2_ map with missing scores are underlined. Vigour QTLs as mapped in BC_1_S_1_ and BC_2_S_1_ families under non-stress (black), drought (red), salt (blue) and nutrient deficiency (green) conditions are shown next to the marker positions. Open QTL block indicates a positive additive effect for the wild allele, and closed QTL block indicates a positive additive effect for the crop allele. Trait abbreviations: L: plant length, FW: fresh weight, DW: dry weight, RMC: relative moisture content.

The linkage maps, shown in Figures 1, 2, 3, 4 and 5, consisted of nine linkage groups (LG) that represented the nine chromosomes of lettuce [32]. The same marker order was obtained in the BC_1 _and BC_2 _populations. The BC_1 _map was made of 347 markers spanning a total length of 1301 cM, while the BC_2 _map had 348 markers with a total length of 1403 cM. Individual linkage groups contained 34 to 50 SNP markers, except LG9, which had 18 markers. As mentioned in the QTL analysis subsection of Materials and Methods, virtual markers were added on the BC_2 _map to fill gaps stemming from the additional round of recombination for better QTL mapping results. These markers are underlined on the BC_2 _linkage map (Figures 1, 2, 3, 4 and 5).

### Quantitative Trait Loci

Twenty QTLs associated with plant vigour were mapped in the BC_1 _population, 5 for plant height, 4 for fresh weight, 4 for dry weight and 7 for relative moisture content (Table 2 and Figures 1, 2, 3, 4 and 5). The QTLs were located on all linkage groups except LG2. Only three of these QTLs had the same order of magnitude additive effect in all treatments. The remaining QTLs were significantly affected by QTLxE.

**Table 2:** Quantitative trait loci mapped in 100 BC_1_S_1 _and 100 BC_2_S_1 _families for vigour traits under non-stress, drought, salt and nutrient deficiency conditions

						Additive effect for the crop allele (% explained variance)				
				
Trait	**Pop**.	QTLname	Most significant marker	LG	QTLxE	**C-D**^**1**^	D	C-SN	N	S
Plant height (cm)	BC_1_	*L-7-1 *	QGF25M24-1	7	yes			-2.06(6)	0.39(3)	
	
		*L-9-1 *	CLS_S3_Contig2201-5-OP5	9	yes			-4.61(32)	-0.79(13)	-2.32(30)
	
		*L-1-1 *	QGC26E22.yg-2-OP4	1	yes				0.74(12)	
	
		*L-3-3 *	QGF21B10.yg.ab1_PAP2_LE1382_12	3	yes		-0.48(6)		0.39(3)	
	
		*L-5-2 *	CLS_S3_Contig1313-2-OP5	5	yes	-1.19(11)		-1.72(4)	-0.49(5)	-1.53(13)

	BC_2_	*L-7-1 *	QGB11B18.yg-2-OP5	7	yes			-1.30(4)		-0.73(3)

		*L-9-1 *	CLS_S3_Contig2201-5-OP5	9	yes					-0.53(3)

		*L-4-4 *	Contig1094-1	4	no	0.42(1)	0.42(6)	0.42(1)	0.42(9)	0.42(2)

		*L-8-2 *	CLX_S3_Contig8250_1298	8	yes	1.46(8)	0.72(14)	1.87(19)	0.38(6)	1.57(24

Fresh weight (g)	BC_1_	*FW-7-2 *	CLSS4482.b2_C18-6-OP5	7	yes	4.02(10)	0.20(4)	0.96(4)	0.34(27)	1.28(22)
	
		*FW-9-2 *	CLS_S3_Contig2201-5-OP5	9	yes	2.38(3)			0.27(17)	0.48(3)
	
		*FW-1-2 *	QGG6E14.yg.ab1_PHYB_1360	1	yes	4.32(11)		1.50(11)	0.14(4)	0.92(11)
	
		*FW-4-5 *	Contig6039-19	4	yes	3.04(6)				
	
	BC_2_	*FW-7-2 *	CLS_S3_Contig7594-1-OP5	7	no	0.28(1)	0.28(9)	0.28(1)	0.28(5)	0.28(1)

		*FW-1-2 *	CLSS3922.b1_C21-4-OP4	1	yes	2.73(7)		2.29(19)		0.97(19)

		*FW-8-1 *	RHCLS_S3_Contig9441_1	8	no	0.32(1)	0.32(13)	0.32(1)	0.32(7)	0.32(2)

Dry weight (g)	BC_1_	*DW-7-3 *	CLSS4482.b2_C18-6-OP5	7	no	0.08(1)	0.08(15)	0.08(3)	0.08(19)	0.08(8)
	
		*DW-4-6 *	CLRY544-1	4	yes	-0.39(16)		-0.04(4)	-0.04(4)	
	
		*DW-8-3 *	QG_CA_Contig5320_RPT3_LE1380_1	8	no	0.05(1)	0.05(6)	0.05(1)	0.05(7)	0.05(3)
	
		*DW-9-6 *	QGG16P08-1	9	yes			-0.09(4)		0.04(6)
	
	BC_2_	*DW-7-3 *	QGF25M24-1	7	no	0.07(1)	0.07(25)	0.07(4)	0.07(12)	0.07(9)
	
		*DW-4-6 *	Contig7363-2	4	yes	-0.21(6)	-0.06(15)	-0.11(8)		

Relative moisture content (%)	BC_1_	*RMC-4-3 *	CLRY544-1	4	yes	0.24(9)	0.60(6)	0.15(4)	1.18(17)	
	
		*RMC-5-1 *	RHCLSM9436.b1_G08_1-OP3	5	yes	0.25(10)				
	
		*RMC-7-4 *	CLSS4482.b2_C18-6-OP5	7	yes		-0.73(9)	-0.17(6)	-0.85(9)	
	
		*RMC-3-4 *	QGF21B10.yg.ab1_PAP2_LE1382_12	3	yes				0.79(8)	0.18(5)
	
		*RMC-4-7 *	CLX_S3_Contig10345_1167_4	4	no	0.22(7)	0.22(1)	0.22(10)	0.22(1)	0.22(4)
	
		*RMC-6-3 *	QGB25B18-1	6	yes				-1.19(17)	

		*RMC-8-4 *	CLS_S3_Contig9218-1-OP5	8	yes		-0.81(11)		0.46(3)	-0.17(4)

	BC_2_	*RMC-4-3 *	Contig15389-1	4	yes	0.32(19)	1.16(14)	0.27(16)	0.89(23)	0.27(9)

		*RMC-7-4 *	QGF25M24-1	7	yes	0.19(7)			-0.56(9)	

		*RMC-1-3 *	CLRX9010-5	1	yes		-1.33(19)	-0.16(5)	-0.60(11)	-0.24(8)

		*RMC-5-3 *	Contig2221-1	5	yes		0.72(6)	-0.21(9)		

1 C-D: control treatment of the drought experiment; D: drought, C-SN: control treatment of the salt-nutrient experiment, N: nutrient deficiency, S: salt

QTLs for plant height had an additive effect positive for the wild allele in the drought and salt treatments, and in the two control treatments. Under nutrient deficiency, two of the plant height QTLs had an additive effect positive for the wild allele, while three QTLs for the same trait were positive for the crop allele, including two QTLs (*L-3-3 *and *L-7-1*) which had a positive effect for the wild allele in other treatments, hence showing opposite allelic effects from one treatment to another.

Fresh weight QTLs were inherited from the crop as three of the QTLs for this trait showed a positive additive effect for the crop allele. Dry weight QTLs were inherited from both the crop and wild parent as three of the QTLs for the trait had a positive additive effect for the crop allele, while one QTL for that trait showed a positive additive effect for the wild allele. Relative moisture content QTLs were inherited from both the crop and the wild parents. Four of the QTLs mapped for this trait had a positive additive effect for the crop allele, while the additive effect was positive for the wild allele for the remaining three QTLs.

Fewer QTLs were mapped in the BC_2 _than in the BC_1 _population (Table 2 and Figures 1, 2, 3, 4 and 5). Thirteen QTLs were mapped in BC_2 _for vigour-related traits. Four of the QTLs were significant in all the treatments with the same additive effect, hence having non-significant QTLxE effect, while the remaining nine were significantly affected by QTLxE.

Two of the QTLs for plant height had a positive additive effect for the wild allele and they were significant under the control treatment of the salt-nutrient experiment and under salt treatment. The other two had a positive additive effect for the crop allele. The three fresh weight QTLs had a positive additive effect for the crop allele. For the dry weight QTLs, one had a positive additive effect from the crop allele and the other one was positive for the wild allele. Relative moisture content QTLs were inherited from both the wild and the crop parent.

### Co-localization of QTL regions

QTL regions on LG4 and LG7 were the most important in the two populations as they comprised most of the QTLs. Four QTLs were mapped on the same region on LG7 in the BC_1 _and BC_2 _populations, one for each of the measured vigour traits (Figure 4). The QTLs for fresh weight and dry weight had the same allelic effect, which was positive for the crop allele under all the treatments. However, the plant height and relative moisture content QTLs showed allelic specificity for treatments in the two populations, with a QTL showing a positive effect from one parent in one treatment and a positive effect for a different treatment from the other parent. On LG4, four QTLs were mapped around the same region in BC_1 _and the same region contained three QTLs in BC_2_, including two QTLs that were common in the two populations. In total 8 QTLs were common in the BC_1 _and BC_2 _populations on LG1, LG4, LG7, and LG9. Additionally, a QTL region was found in both populations on LG8 but it contained QTLs for different traits in the two populations.

### QTL epistatic effects

QTL epistatic effects on the vigour traits were significant in the two hybrid populations and under stress and non-stress conditions. In the BC_1 _population epistasis was estimated for 10 QTL pairs and it explained 4 to 9% of the phenotypic variance per individual QTL pair and up to 23% per trait. Nine QTL regions were used in the BC_2 _population, supplemented with 6 QTL regions that, on their own, were only significant in the BC_1_, in order to increase the number of loci in the epistasis analysis. Epistasis for these regions explained 3 to 11% of the phenotypic variance per QTL pair and up to 27% per trait (Table 3). While interacting QTLs for plant height had a higher mean for the crop-crop or wild-wild genotype combinations in the BC_1 _population, the highest mean for the same trait was associated with crop-wild genotype combinations in the BC_2 _population, showing the effect of the combination of QTLs inherited from the two parents for that trait. The genotype combination of a wild allele at the two epistatic loci (b/b) was associated with the highest mean for 3 out of 17 QTL pairs in BC_1 _and 3 out of 23 QTL pairs in BC_2_, indicating that the advantageous epistatic effect was mostly associated with the genotype combinations involving a crop allele at one of the two loci.

**Table 3:** Significant QTL × QTL interactions in the BC_1 _and BC_2 _populations, their explained phenotypic variance and the predicted means per genotype combination

				**% expl. variance Predicted mean per genotype combination**^**1**^			
**Population**	**Treat**.	**Trait**	**QTLxQTL**	**h/h**	**h/b**	**b/h**	**b/b**

BC_1_	Control-D	Plant height	*L-1-1 × DW-4-6 *5	30.71	30.30	30.54	31.86

		Dry weight	*L-1-1 × RMC-8-4 *4	2.947	3.10	2.832	2.578

		Relative moisture content	*L-7-1 × L-5-2 *4	93.98	93.74	93.84	93.98
		
			*L-7-1 × RMC-5-1 *5	94.09	93.62	93.96	93.89
		
			*L-1-1 × L-5-2 *4	93.77	93.95	94.00	93.8

	Control-SN	Plant height	*L-9-1 × RMC-5-1 *4	26.14	27.20	29.29	33.79
		
			*L-3-3 × RMC-8-4 *5	29.76	28.49	27.46	29.94

		Relative moisture content	*L-1-1 × L-5-2 *7	92.18	92.40	92.30	92.11

	Salt	Plant height	*L-5-2 × RMC-8-4 *4	17.85	16.61	17.01	17.68

		Fresh weight	*DW-9-6 × RMC-5-1 *5	11.44	10.78	10.38	11.06

		Relative moisture content	*L-1-1 × L-5-2 *7	90.07	90.34	90.25	90.02
		
			*L-3-3 × DW-8-3 *7	90.05	90.39	90.17	90.05

			*L-3-3 × RMC-8-4 *9	90.09	90.41	90.20	90.04

	Nutrient deficiency	Plant height	*L-3-3 × RMC-8-4 *4	10.56	10.19	9.62	10.16
		
			*DW-8-4 × RMC-5-1 *5	9.85	10.41	9.18	10.79
		
		Dry weight	*L-9-1 × DW-8-3 *4	0.67	0.59	0.58	0.58

		Relative moisture content	*DW-8-3 × DW-9-6 *5	80.17	80.87	81.53	80.86

BC_2_	Control-D	Plant height	*L-7-1 × L-8-2 *5	28.87	29.04	31.06	28.69

		Fresh weight	*FW-1-2 × DW-9-6 *5	37.80	40.99	41.41	37.33

			*L-3-3 × RMC-6-3 *8	29.61	40.00	39.01	38.89

			*L-7-1 × L-3-3 *8	33.83	39.58	41.74	38.49

		Relative moisture content	*L-9-1 × DW-4-6 *11	93.43	92.82	92.82	92.77

			*L-4-4 × L-8-2 *4	92.77	93.04	92.85	92.78

	Drought	Plant height	*L-7-1 × L-8-2 *5	14.42	14.29	15.11	14.02

	Control-SN	Plant height	*L-7-1 × L-8-2 *5	21.20	20.69	23.71	21.16
		
		Fresh weight	*L-4-4 × DW-9-6 *8	19.65	17.22	16.97	17.76

			*L-3-3 × RMC-6-3 *7	14.89	18.04	17.94	17.70

		Dry weight	*L-4-4 × DW-9-6 *8	1.46	1.26	1.28	1.32

			*L-3-3 × RMC-6-3 *3	1.12	1.33	1.34	1.31

		Relative moisture content	*L-9-1 × DW-4-6 *7	93.09	92.56	92.64	92.54

	Salt	Plant height	*L-9-1 × RMC-5-3 *5	16.91	16.04	16.11	16.98

		Fresh weight	*DW-4-6 × L-5-2 *4	11.50	10.34	9.88	10.44

		Dry weight	*L-8-2 × FW-1-2 *5	0.93	0.95	0.98	0.87

	Nutrient deficiency	Plant height	*L-7-1 × FW-1-2 *6	10.95	11.37	11.68	11.26
		
		Fresh weight	*L-7-1 × FW-1-2 *6	5.42	5.70	5.47	5.06
		
			*RMC-1-3 × RMC-5-3 *5	5.73	5.20	5.19	5.41

		Relative moisture content	*L-9-1 × DW-4-6 *6	87.87	86.45	86.84	86.56

			*DW-4-6 × RMC-1-3 *6	86.01	87.65	86.24	86.63

			*L-7-1 × RMC-1-3 *6	85.54	86.78	86.79	86.97

			*FW-1-2 × DW-9-6 *8	86.76	86.53	86.13	86.92

1 h: heterozygous genotype, b: homozygous for the wild allele

## Discussion

### Performance of crop-wild hybrid lines

Studies on introgression of crop genes into wild relative genomes have shown that although the average fitness of the hybrids might be lower than the fitness of the wild relative, individual hybrid genotypes could have similar or better fitness than their wild parent, showing a potential for introgression of advantageous crop genes [33,34]. In our study, the BC_1_S_1 _and BC_2_S_1 _families revealed lines showing transgressive segregation for vigour in the control and stress treatments, indicating that two generations of backcrossing to the wild parent did not eliminate the effect of the crop segments. The occurrence of BC_2_S_1 _families that outperform the wild parent shows that if vigour traits positively correlate with fitness under natural conditions, crop genomic segments that confer improved vigour could be introgressed into the wild taxon, rendering it more vigorous under non-stress as well as under abiotic stress conditions.

### QTL effects

Backcrossing has been applied in plant breeding for fine-mapping of QTLs and for the introgression of desired QTL alleles from wild donors into elite cultivars [35-37]. In crop-to-wild gene flow, repeated backcrossing to the wild parent might take place along with selfing as a result of the often much higher frequency of wild individuals compared to crop-wild hybrids. One of the direct consequences of repeated backcrossing to the wild species is the continuing decrease in crop genomic segments, both in size as they become successively shorter and in frequency as each plant has fewer segments. Consequently, each backcrossing event is expected to reduce the detection power of QTL analysis [38]. Consistently with this, we detected more QTLs in the BC_1 _population than in the BC_2 _population for each of the considered vigour traits. However, despite the decreasing crop content, new QTLs with an additive effect from the crop allele were detected in the backcross populations, including the BC_2_, as compared to the F_2_, which we have studied in earlier work [Uwimana *et al. *submitted]. In the F_2 _study [Uwimana *et al. *submitted], plant height QTLs in the F_2 _population were entirely inherited from the wild parent. In this study, two additional plant height QTLs were mapped for the nutrient deficiency treatment (*L-1-1 *and *L-3-3*) in the BC_1 _population with an additive effect from the crop. In the BC_2 _population we detected two more QTLs for plant height (*L-4-4 *and *L-8-2*) with the same allelic effect in all the treatments which was positive for the crop allele, showing that the contribution of the crop to plant vigour could be underestimated depending on the population studied.

### Common QTLs in selfing and backcrossing hybrid generations

We found QTL regions related to vigour under control and three abiotic stress conditions, showing a diverse potential introgression mosaic with contributions of genomic segments from both crop and wild relative parents. Many of these QTLs co-localised, allowing to pinpoint introgression "hotspots". Seven QTLs were common between F_2_, BC_1 _and BC_2 _populations on LG4, LG7 and LG9, three were common in at least two populations on LG1 and LG5, and one QTL was found in very closely located regions in the backcross populations on LG4 (Table 4). A common finding in plant breeding is that different QTLs are detected in different mapping populations of the same cross. The differences could be attributed to statistical power, especially with a limited number of lines in the population (< 200), and to a combination of recessiveness and a skewed linkage map [39]. Differences in detected QTLs between populations has also been associated with changes in genetic variation between populations with further backcrossing associated with decreasing genetic variation and consequently resulting in decreasing QTL detection power [38]. In the present study, the QTLs common to more than one hybrid generation were those with the greatest effects in terms of explained phenotypic variance per treatment and per trait, while the QTLs with small effect were mostly mapped in one hybrid generation.

**Table 4:** Recapitulation on common QTLs for vigour in the three hybrid populations F_2_, BC_1 _and BC_2 _under non-stress (C), drought (D), salt (S) and nutrient deficiency (N) conditions

			**F**_**2**_				**BC**_**1**_				**BC**_**2**_			
**Trait**	**QTL**	**LG**	**C**	**D**	**N**	**S**	**C**	**D**	**N**	**S**	**C**	**D**	**N**	**S**

Plant height	*L-7-1*	7	-		-	-	-		+		-			-
	
	*L-9-1*	9	-		-	-	-		-	-				-

Fresh weight	*FW-7-2*	7		+	+	+	+	+	+	+	+	+	+	+
	
	*FW-8-1*	8	+		-	+				+		+	+	+
	
	*FW-9-2*	9				+	+		+	+				
	
	FW-1-2	1					+		+	+	+			+

Dry weight	*DW-7-3*	7	-	+			+	+	+	+	+	+	+	+
	
	*DW-4-6*	4					-		-		-	-		

Relative moisture content	*RMC-4-3*	4	+	+	+		+	+	+	+		+	+	+
	
	*RMC-5-1*	5	+	+			+							
	
	*RMC-7-4*	7	+		-	+	-	-	-		+		-	

The positive sign shows a positive effect for the crop allele and the negative sign shown a positive effect for the wild allele

Linkage groups 4, 7 and 9 were the most important in BC_1 _and BC_2 _populations as they showed regions that contained many and common QTLs in the two populations. The same regions were important in the F_2 _population [Uwimana *et al. *submitted]. Despite the overlapping QTL regions across hybrid populations, some QTLs showed treatment specificity per population. For instance, *L-7-1 *had a positive effect for the wild allele under nutrient deficiency conditions in the F_2 _population, but the same QTL region showed a positive effect for the crop allele under the same treatment in the BC_1 _population and it was not significant in the BC_2 _population. Conversely, *RMC-4-3 *was consistent across populations and treatments with a positive allelic effect from the crop, though it was not significant in the salt treatment of the F_2 _population. Such QTLxE interactions suggest that the regions might contain different treatment-specific genes which contribute to the vigour of the plants. Moreover, QTLs for different vigour traits were mapped in those same regions with opposite allelic effect. Nevertheless, the involvement of the same regions in the vigour of the hybrids in three populations indicates that these regions will be under selection, either positive or negative, depending on the prevailing conditions.

### Epistasis

QTL epistatic effect was significant for several vigour traits in the two backcross populations. Epistasis has been suggested as one of the major allelic interactions affecting fitness in self-pollinating species such as *Arabidopsis thaliana *[40] and rice [41]. Epistatic QTL effects are expected to play a major role in selfing populations and to decline with further backcrossing as a result of decreasing genetic variation [38]. Our results show that the vigour traits were affected by the epistatic effect of QTLs under stress and non-stress conditions, and that positive epistatic effects were mostly associated with genotype combinations involving the crop alleles. QTL epistatic effect in BC_1 _and BC_2 _populations emphasizes the genetic importance of the crop genomic segments even after two backcrosses to the wild parent. Importantly, the combination of beneficial epistatic and additive allelic effects from two parents at different loci in repulsion phase has been associated with the origin of transgressive segregation that leads to the creation of superior or even ecologically diverging phenotypes [12,42,43]. However, the fact that none of the QTL epistatic effects were detected in both populations makes the stability of the epistatic effect over generations questionable; in turn, this will make it difficult to predict the effect in further generations.

## Conclusions

Both in the BC_1 _and BC_2_, lines were identified that performed equally or better than both the wild parental genotype and the additional wild genotype included in the experiments, indicating the occurrence of transgressive segregation in our hybrid populations. Epistasis may be an important underlying factor and some positive epistatic effects of QTLs were detected, mostly associated with crop alleles, but these were not universal across F_2 _and BC generations. Knowledge of fitness effects of crop genomic blocks (QTLs) may be put to use to control (trans)gene flow to natural populations, namely by inserting genes that one would prefer to keep contained, into genomic regions disadvantageous to the plant's performance in the field. Although fewer QTLs were detected in the BC_2 _than in the BC_1 _and there was also some variation in QTLs between the F_2 _and the BC generations, many QTLs were found to be in common between hybrid generations. Among these, there were QTLs for which the crop alleles were clearly disadvantageous to the plant's performance and QTLs for which the crop alleles were advantageous. The latter genomic areas would not be advisable for inserting genes to be contained. It was also possible to identify "hotspots" of QTLs, which would also better be avoided as they are clearly important to the plant's performance and mostly show advantageous as well as disadvantageous effects for the respective crop alleles. This study was carried out on plant vigour, based on the previous knowledge that lettuce crop-wild hybrids undergo selection at an early stage of growth [9]. To our knowledge, this is the first study on introgression that combines a QTL analysis approach with different stress treatments to address the process of introgression. Although these experiments were conducted using a limited number of hybrid genotypes (the hybrids were derived from a cross between single crop and wild genotypes) and under greenhouse conditions, the results constitute a first, informative step towards understanding the potential for introgression of cultivated lettuce genomic segments into wild lettuce under abiotic stress conditions. Future experiments should consider the whole life cycle of hybrid plants from seed germination to seed production under field conditions, as to include early and late plant vigour, natural selection and survival, and reproduction as well as a greater range of crop and wild genotypes.

## Methods

### Creation of BC_1 _and BC_2 _hybrid progenies and genotyping

The present study concerns two backcross populations, BC_1 _and BC_2_, back-crossed to *L. serriola *to mimic an important pathway for natural introgression from a crop to its wild relative. Flowers from the F_1 _hybrid plant resulting from a cross between *L. serriola *(collected from Eys, the Netherlands) [44], and *L. sativa *(cv. Dynamite) were hand-pollinated with pollen from the *L. serriola *parental line to generate BC_1 _plants according to the lettuce pollination protocols by Nagata [45] and Ryder [46]. By the same method, BC_2 _plants were created using the same *L. serriola *parental line.

The Compositae Genome Project has developed 1083 Single Nucleotide Polymorphism (SNP) markers in lettuce from disease resistance and developmental genes http://compgenomics.ucdavis.edu/compositae_SNP.php. From the 1083 SNPs, a customised Illumina GoldenGate array of 384 SNPs was developed specifically for the markers showing polymorphism between the parents used in our crop-wild cross and with approximately even genetic distribution [Uwimana *et al. *submitted]. A set of 192 BC_1 _individuals were genotyped using the 384 SNP custom array. Based on their genotypes, 100 BC_1 _individuals were selected that optimized the genetic diversity of the population using the program "Genetic Distance Optimization program" (GDOpt), [47]; and these were used in greenhouse experiments. Forty-five of the 100 BC_1 _plants were backcrossed to *L. serriola *to generate BC_2 _lines. At the same time, the BC_1 _lines were left to self-pollinate to BC_1_S_1 _seeds (Additional file 1: Figure S1). Six hundred BC_2 _individuals (12 BC_2 _plants for each of the 45 back-crossed BC_1 _individuals) were selfed to produce BC_2_S_1 _seeds. Four hundred fifty-eight BC_2 _individuals were randomly selected and genotyped with the customized 384 SNP array. Based on their genotypes, a selection of 100 BC_2 _individuals was also made using the program GDOpt and their BC_2_S_1 _progenies were used in greenhouse experiments.

### Greenhouse experiments

The BC_1_S_1 _and BC_2_S_1 _seeds of the selected 100 BC_1 _and 100 BC_2 _individuals were used in greenhouse experiments together with their parents (*L. serriola*/Eys and *L. sativa *cv. Dynamite). We also included two lines, *L. serriola *acc. UC96US23 and *L. sativa *cv. Salinas (parents of the reference RIL population used in various experiments [48,49]), which, together with the parents of our population, were used to estimate the environmental error. Our parents and the two additional lines were replicated 12 times per treatment and each BC_1 _and BC_2 _individual was represented by 12 BC_1_S_1 _and BC_2_S_1 _seedlings, respectively, per treatment.

Experiments were conducted separately for the BC_1 _and BC_2 _populations, using the same set as in the F_2 _experiments [Uwimana *et al. *submitted]. For each population, two experiments were carried out, one comprising salt and nutrient treatments together with a control treatment and another experiment comprising a drought treatment together with a control treatment. The drought experiment for the BC_1 _population was carried out in the period of February-March 2009, the salt and nutrient experiment for the BC_1 _population was carried out in April-May 2009, the drought experiment for the BC_2 _population was carried out in November 2009-January 2010 and the salt and nutrient experiment of the same population was carried out in January-March 2010. After transplanting, the plants were watered twice a week with 1.3 EC nutrient solution for two weeks, after which the stress treatments were applied. For the drought treatment, the plants were not given water for three weeks; for salt treatment, irrigation nutrient solution was supplemented with 100 mM NaCl, and for nutrient deficiency treatment, plants were irrigated with water without nutrients for three weeks. At the end of the fifth week after transplanting (at the rosette stage) we measured plant vigour for individual plants as shoot height, shoot fresh weight and shoot dry weight (after drying at 80°C for 3 days). We calculated shoot relative moisture content as the ratio of the amount of water in the shoot to the total shoot weight [(fresh weight-dry weight)*100/fresh weight].

### Construction of the linkage maps

Out of 384 SNP markers, 347 were successfully scored in the 100 BC_1 _individuals and 348 in the 458 BC_2 _individuals. Genetic linkage maps of the two populations were built separately using JoinMap^® ^4 [50]. The marker grouping into linkage groups in the BC_2 _population was kept the same as in the BC_1 _and F_2 _populations, and the order of the markers and their genetic distances were calculated based on recombination among the BC_2 _individuals. The linkage maps were displayed using MapChart 2.2 [51].

### Analysis of phenotypic data

Statistical analysis was performed using GenStat 14th Edition [52]. The drought and the salt-nutrient experiments were analysed separately for each of the BC_1 _and BC_2 _populations. The significance of the different terms was determined by the analysis of variance, fitting the model *Response = general mean + block + genotype + treatment + genotype.treatment + error*; with the term *genotype *representing the hybrid families (100 × 12 BC_1_S_1 _or 100 × 12 BC_2_S_1_). For the QTL analysis, broad sense heritability of family means of the traits in each of the experimental populations was estimated for each treatment as the proportion of the total variance accounted for by the genetic variance using the formula:

$$H^{2}=Vg/\left(Vg+Ve/r\right);$$

where *Vg *is the genetic variance for the BC_1_S_1 _or BC_2_S_1 _families, *Ve *is the environmental variance, and *r *is the number of replications [53]. *Vg *was estimated based on the restricted maximum likelihood (REML) method from the mixed model:

Response=general mean+block+genotype+error;

with the term *genotype *taken random. Because BC_1_S_1 _and BC_2_S_2 _families were segregating, the term *Ve *was the error variance derived from a one-way analysis of variance of the model:

Response=general mean+block+parents+error;

with the term *parents *representing the two parents (*L. serriola*/Eys and *L. sativa *cv. Dynamite) and the two added lines (*L. serriola *acc. UC96US23 and *L. sativa *cv. Salinas).

### Quantitative Trait Loci analysis

The genetic linkage map, the genotype scores and the phenotypic means were combined for QTL analysis using the QTL analysis function of GenStat 14th Edition [52]. To adjust for the calculation differences caused by the marker gaps due to the additional recombination event in the BC_2 _population, the gaps in the BC_2 _linkage map were filled with virtual markers which were given missing marker scores. Thirty-five virtual markers were added on seven linkage groups (LG1, 2, 3, 4, 5, 8 and 9), keeping a maximum distance of 12 cM between the markers (Figures 1, 2, 3, 4 and 5). This resulted in the best estimate of a QTL region, but the presence of QTLs in the BC_1 _and BC _2 _populations were analyzed separately.

In order to effectively model genotype by environment interaction (GxE, with environments represented by the different treatments) through QTL by environment interaction (QTLxE), each trait was analysed individually using the single trait - multiple environment option of the program. Genome-wide association between markers and traits was decided based on a significance level of 0.05 corrected for multiple tests using the Li and Ji method [54]. After the selection of the best variance-covariance model for the treatments [55], the candidate QTLs were determined by initial genome scan. Final QTL positions were determined by composite interval mapping taking into account co-factors. The allelic effect of the detected QTLs in each treatment, the effect of QTLxE and the explained phenotypic variance of each QTL per treatment were determined by running a backward selection on the candidate QTLs in a mixed linear model, taking the QTL effect in each treatment as fixed terms and the interaction between each hybrid family and the treatment as random [56]. In that way, each QTL detected in one treatment was tested for its effect and significance in the other treatments.

To test for QTL epistatic effect (QTL × QTL), the phenotypic means were regressed against the genotypes of the most significant markers for each QTL in a generalized linear model. One marker was considered for each QTL region, and no QTL interaction was estimated for QTLs on the same LG. For each treatment, every trait was explained by the main effects of all the detected QTLs to which interaction between one pair of QTLs was added at a time. The interaction effects of the QTL regions that were significant in the BC_1 _population were also included in the QTLxQTL analysis in BC_2_. QTLxQTL interaction was decided significant at a level of 0.05 corrected for the number of the traits using the Bonferroni method [57].

## Authors' contributions

BU conducted this work as part of her PhD thesis. She contributed to the creation of the hybrids and the design of the study. She carried out the experiments, collected the data, performed statistical analysis, linkage mapping and QTL analyses and drafted the manuscript. MJMS, CCMvdW, PHvT and RGFV conceived and designed the study and supervised the work. DAPH conceived and designed the study and generated the hybrids. YH generated the hybrids and participated in the design of the experiments. JJ contributed to statistical and QTL analysis. LKMcH and RWM developed the SNP markers and were involved in genotyping the samples. All authors critically reviewed the manuscript. All authors read and approved the final manuscript.

## Supplementary Material

Additional file 1 **Below is the link to the electronic supplementary material. Figure S1**. Crossing and experimental scheme of the study on introgression process from cultivated to wild lettuce. The backcrossing pathway (BC_1 _and BC_2 _populations) is the subject of this study. Figure S2. Allelic composition of the selected BC_1 _(A) and BC_2 _(B) genotypes. Blue: homozygous for the wild allele; yellow: hetereozygous; black: missing genotype scores. Backcrossing to the wild parent reduces the crop genome content in amount and in segment size. Figure S3. Boxplots representing the phenotypic variation among BC_1_S_1 _relative to *L. serriola *acc. UC96US23 (P1), *L. sativa *cv. Salinas (P2), *L. serriola/*Eys (P3) and *L. sativa *cv. Dynamite (P4) for vigour traits dry weight (A), fresh weight (B), plant height (C) and relative moisture content (D) under the five treatments. Figure S4. Boxplots representing the phenotypic variation among BC_2_S_1 _plants relative to *L. serriola *acc. UC96US23 (P1), *L. sativa *cv. Salinas (P2), *L. serriola/*Eys (P3) and *L. sativa *cv. Dynamite (P4) for vigour traits dry weight (A), fresh weight (B), plant height (C) and relative moisture content (D) under the five treatments. Click here for file
