# Supplementary material for: Crop to wild introgression in lettuce: following the fate of crop genome segments in backcross populations
Source: BMC Plant Biol. 2012 Mar 26;12:43. doi: 10.1186/1471-2229-12-43 (PMC3384248; doi:10.1186/1471-2229-12-43)
Supplement: Additional file 1 — Below is the link to the electronic supplementary material. Figure S1. Crossing and experimental scheme of the study on introgression process from cultivated to wild lettuce. The backcrossing pathway (BC1 and BC2 populations) is the subject of this study. Figure S2. Allelic composition of the selected BC1 (A) and BC2 (B) genotypes. Blue: homozygous for the wild allele; yellow: hetereozygous; black: missing genotype scores. Backcrossing to the wild parent reduces the crop genome content in amount and in segment size. Figure S3. Boxplots representing the phenotypic variation among BC1S1 relative to L. serriola acc. UC96US23 (P1), L. sativa cv. Salinas (P2), L. serriola/Eys (P3) and L. sativa cv. Dynamite (P4) for vigour traits dry weight (A), fresh weight (B), plant height (C) and relative moisture content (D) under the five treatments. Figure S4. Boxplots representing the phenotypic variation among BC2S1 plants relative to L. serriola acc. UC96US23 (P1), L. sativa cv. Salinas (P2), L. serriola/Eys (P3) and L. sativa cv. Dynamite (P4) for vigour traits dry weight (A), fresh weight (B), plant height (C) and relative moisture content (D) under the five treatments. [file 1471-2229-12-43-S1.DOCX]

**Supplementary material**

100 x 12

45 x 12

100 x 12

100 x 12

Dynamite

BC1

BC1S1

BC2

BC2S1

F_1_

Expected average % crop 12.5 25 50 100

F1S1

F1S2

Genotyping

Experiments

BC_1_

BC_1_S_1_

BC_2_

BC_2_S_1_

F_2_

F_2:3_

*L. sativa* (cv. Dynamite)

*L. serriola* (Eys)

Figure S1 Crossing and experimental scheme of the study on introgression process from cultivated to wild lettuce. The back-crossing pathway (BC_1_ and BC_2_ populations) is the subject of this study

**B**

**A**

Genotypes

**Figure S2** Allelic composition of the selected BC_1_ (A) and BC_2_ (B) genotypes. Blue: homozygous for the wild allele; yellow: hetereozygous; black: missing genotype scores. Backcrossing to the wild parent reduces the crop genome content in amount and segment size

**Figure S3** Boxplots representing the phenotypic variation among BC_1_S_1_ (position 1) relative to *L. serriola* acc. UC96US23 (position 2), *L. sativa* cv. Salinas (position 3), *L. serriola/*Eys (position 4) and *L. sativa* cv. Dynamite (position 5) for vigour traits dry weight (A), fresh weight (B), plant height (C)and relative moisture content (D) under the five treatments

**Drought**

**Control_Drought**

**Nutrient deficiency**

**Control_Salt-Nutrient**

**Salt**

**D**

**C**

**B**

**A**

Figure S4 Boxplots representing the phenotypic variation among BC_1_S_1_ and BC_2_S_1_ plants (position 1) relative to *L. serriola* acc. UC96US23 (position 2), *L. sativa* cv. Salinas (position 3), *L. serriola/*Eys (position 4) and *L. sativa* cv. Dynamite (position 5) for vigour traits dry weight (A), fresh weight (B), plant height (C)and relative moisture content (D) under the five treatments

**A**

**B**

**C**

**D**

**Control_Drought**

**Drought**

**Nutrient deficiency**

**Control_Salt-Nutrient**

**Salt**
